# Reduction of Leukocyte Microvascular Adherence and Preservation of Blood-Brain Barrier Function by Superoxide-Lowering Therapies in a Piglet Model of Neonatal Asphyxia

**DOI:** 10.3389/fneur.2019.00447

**Published:** 2019-05-01

**Authors:** Jacob B. Ruden, Kevin L. Quick, Ernesto R. Gonzales, Aarti R. Shah, T. S. Park, Nan Kennedy, Laura L. Dugan, Jeffrey M. Gidday

**Affiliations:** ^1^Vanderbilt Brain Institute, Vanderbilt University, Nashville, TN, United States; ^2^PerkinElmer, Waltham, MA, United States; ^3^Hope Center for Neurological Disorders and Department of Neurology, Washington University in Saint Louis School of Medicine, St. Louis, MO, United States; ^4^Knight Alzheimer's Disease Research Center, Washington University in Saint Louis School of Medicine, St. Louis, MO, United States; ^5^Department of Neurosurgery, St. Louis Children's Hospital, Washington University in Saint Louis School of Medicine, St. Louis, MO, United States; ^6^Vanderbilt Institute for Clinical and Translational Research, Vanderbilt University Medical Center, Nashville, TN, United States; ^7^Division of Geriatric Medicine, Department of Medicine, Vanderbilt University Medical Center, Nashville, TN, United States; ^8^Departments of Ophthalmology, Physiology, and Neuroscience, Louisiana State University School of Medicine, New Orleans, LA, United States

**Keywords:** asphyxia, carboxyfullerene, endothelium, inflammation, leukocytes, oxypurinol, superoxide, xanthine oxidase

## Abstract

**Background:** Asphyxia is the most common cause of brain damage in newborns. Substantial evidence indicates that leukocyte recruitment in the cerebral vasculature during asphyxia contributes to this damage. We tested the hypothesis that superoxide radical (${\text{O}}_{2}^{\underline{\cdot }}$) promotes an acute post-asphyxial inflammatory response and blood-brain barrier (BBB) breakdown. We investigated the effects of removing ${\text{O}}_{2}^{\underline{\cdot }}$ by superoxide dismutase (SOD) or C_3_, the cell-permeable SOD mimetic, in protecting against asphyxia-related leukocyte recruitment. We also tested the hypothesis that xanthine oxidase activity is one source of this radical.

**Methods:** Anesthetized piglets were tracheostomized, ventilated, and equipped with closed cranial windows for the assessment of post-asphyxial rhodamine 6G-labeled leukocyte-endothelial adherence and microvascular permeability to sodium fluorescein in cortical venules. Asphyxia was induced by discontinuing ventilation. SOD and C_3_ were administered by cortical superfusion. The xanthine oxidase inhibitor oxypurinol was administered intravenously.

**Results:** Leukocyte-venular adherence significantly increased during the initial 2 h of post-asphyxial reperfusion. BBB permeability was also elevated relative to non-asphyxial controls. Inhibition of ${\text{O}}_{2}^{\underline{\cdot }}$ production by oxypurinol, or elimination of ${\text{O}}_{2}^{\underline{\cdot }}$ by SOD or C_3_, significantly reduced rhodamine 6G-labeled leukocyte-endothelial adherence and improved BBB integrity, as measured by sodium fluorescein leak from cerebral microvessels.

**Conclusion:** Using three different strategies to either prevent formation or enhance elimination of ${\text{O}}_{2}^{\underline{\cdot }}$ during the post-asphyxial period, we saw both reduced leukocyte adherence and preserved BBB function with treatment. These findings suggest that agents which lower ${\text{O}}_{2}^{\underline{\cdot }}$ in brain may be attractive new therapeutic interventions for the protection of the neonatal brain following asphyxia.

## Introduction

Asphyxia is a relatively common source of neonatal brain damage (1), affecting ~2 in every 1,000 births (2). The hypoxic ischemia resulting from this oxygen deprivation can produce long-term sequelae, including intellectual disability and neurologic deficits, and nearly a quarter of neonatal deaths worldwide are attributable to birth asphyxia (2). A dominant response to ischemia is inflammation (2, 3), resulting from the accumulation of circulating leukocytes in the cerebral microvasculature and their subsequent extravasation into brain parenchyma (4–7). The mechanisms promoting the early increase in leukocyte-endothelial interactions in the cerebral circulation await further elucidation, but probable causes include the elaboration of cytokines and chemokines (8, 9), and other proinflammatory mediators (10). There is also consensus that reactive oxygen species (ROS), formed during reperfusion following transient ischemia, including in the neonatal brain, also modulate inflammation (11–13).

ROS are generated in multiple compartments within a cell and are produced by many sources, including mitochondria, peroxisomal lipid metabolism, NADPH oxidases, cyclooxygenase, and xanthine oxidase (14–16). Increased levels of ROS are involved in the pathophysiology of many neurodegenerative diseases, stroke, aging, and birth asphyxia (13, 17–20). *In vitro*, ${\text{O}}_{2}^{\underline{\cdot }}$ is increased in neurons (21) and cerebral endothelial cells (22) in response to anoxia-reoxygenation stimuli that simulate ischemia-reperfusion. Activated leukocytes can form copious amounts of ${\text{O}}_{2}^{\underline{\cdot }}$ from NADPH oxidase during their respiratory burst (23). Xanthine oxidase and cyclooxygenase are two enzymatic sources of ${\text{O}}_{2}^{\underline{\cdot }}$, in addition to formation of the radical in the electron transport chain of mitochondria (23, 24).

Considerable evidence implicates ROS in playing a causal role in ischemic brain damage (12, 23, 25). A burst of ${\text{O}}_{2}^{\underline{\cdot }}$ during reoxygenation has been suggested to cause direct cellular damage to macromolecules and initiate cellular signaling pathways involved in cell survival and death, resulting in apoptosis in ischemic lesions. The neonatal brain is believed to be especially vulnerable to free radical damage, due in part to its low amount of antioxidants (1).

Although a number of therapies are under consideration for treating moderate to severe neonatal asphyxia (2, 26), therapeutic hypothermia currently remains the standard treatment, despite evidence showing that the long-range neuroprotective benefits of hypothermia treatment are modest (1). A new potential treatment for neonatal asphyxia is C_3_, a small, cell-permeable C_60_ fullerene compound with SOD mimetic properties: *e,e,e* tris malonic acid (*e,e,e*-C_60_(C(COOH_2_))_3_). Due to its arrangement of malonic acid groups, C_3_ is amphiphilic and cell-permeable, and can catalytically remove ${\text{O}}_{2}^{\underline{\cdot }}$ (27). C_3_ protected against ischemia-mediated release of the intracellular enzyme lactate dehydrogenase in mouse retinal endothelial cells (28). Additionally, C_3_ improved motor function and protected against striatal injury in parkinsonian non-human primates, a model known to involve inflammation (29). C_3_ has proven to be neuroprotective in other injury models (30, 31) and to improve cognitive function and survival in a murine model of aging (19). In contrast to these cytoprotective effects, the effects of C_3_ on inflammatory endpoints are unknown.

In the present study, we utilized SOD, C_3_, and the xanthine oxidase inhibitor oxypurinol to examine the involvement of ${\text{O}}_{2}^{\underline{\cdot }}$ in mediating leukocyte adherence to post-capillary cerebral venules and BBB permeability changes in response to asphyxia. We tested whether ${\text{O}}_{2}^{\underline{\cdot }}$ is produced in response to asphyxia, whether xanthine oxidase is a source of this radical, and whether the inflammatory response caused by asphyxia can be prevented by removing ${\text{O}}_{2}^{\underline{\cdot }}$ by SOD or C_3_ administration, or by preventing the formation of this radical.

## Methods

### Ethical Approval

Washington University Institutional Animal Care and Use Committee approved the experiments, which were consistent with Public Health Service guidelines. All experiments were conducted in compliance with the ARRIVE guidelines.

### Animals

Experiments were performed on piglets that were 1–4 days of age, 1.5–3.0 kg, and of mixed sex. Animals were randomized into control, asphyxia, and asphyxia plus treatment groups. Fluorescently-labeled leukocytes within pial microvessels on the cortical surface of the piglet brain were imaged through a closed cranial window. Rhodamine 6G was used to label circulating leukocytes *in situ* (loading dose: 2 ml/kg of a filtered 0.06 mg/ml solution, 20 min prior to the first baseline measurement; Sigma Chemical, St. Louis, MO), as previously described (32–34). To enhance leukocyte labeling, additional rhodamine was infused (800 μl/min/kg for 30–45 s) before each imaging time point. Real-time, high-resolution images of rhodamine-labeled leukocytes were recorded to videotape (Super VHS) using an epifluorescence microscope (Olympus, Lake Success, NY) with a rhodamine-specific optical filter (535 nm/35 nm excitation, 565 nm dichroic, 610 nm/75 nm emission), 10 × immersion lens (0.4 numerical aperture, 3.1 mm working distance; Olympus), and a C-2400 Newvicon tube camera (Hamamatsu, Bridgewater, NJ).

Piglets were anesthetized with ketamine hydrochloride (20 mg/kg IM), tracheostomized, and ventilated with room air and oxygen. End-tidal CO_2_ and transcutaneous O_2_ were monitored continuously using a capnometer and forepaw sensor, respectively. Anesthesia was maintained by ventilating with isoflurane (1.0–1.5%) while paralysis was sustained using pancuronium (0.25 mg/kg/h IV). A cannula was placed in one femoral vein for administration of the paralytic agent and 5% dextrose in 0.45% normal saline (6 ml/kg/h) to maintain fluid status. Both femoral arteries were cannulated to record arterial blood pressure and to measure blood gases, blood glucose, and pH. Body temperature was maintained at 38–39°C using a thermoregulated heating pad. A closed Plexiglass cranial window was mounted over the right parietal cortex just posterior to the coronal suture and was slowly filled with artificial cerebral spinal fluid (aCSF) buffer after mounting. Through circumferentially placed ports on the window, intracranial pressure was monitored.

### Asphyxia

After the cranial window was placed, the animal and surgical site were allowed to stabilize for 30 min, and then two baseline video recordings were obtained 30 min apart. Asphyxia was then induced by discontinuing ventilation for 9 min, resulting in severe hypoxia, hypercapnia, hypotension, bradycardia, and acidosis. These conditions resolved in the vast majority of animals within 30 min after resuming ventilation without the use of resuscitation (32–34). A very small minority of animals died in response to asphyxia. Arterial blood pressure was recorded and blood gases, blood glucose, and pH were measured at baseline and at 1 and 2 h of reperfusion.

In this study, C_3_ and SOD were administered by cortical superfusion through the cranial window, whereas oxypurinol was given intravenously. To determine whether window superfusion of aCSF affected leukocyte adhesion differently than allowing aCSF to remain static, we established two asphyxia groups. In one, the cranial window was filled with aCSF buffer before baseline measurements were initiated, and the fluid beneath the windows remained undisturbed. In another, the window was superfused at 50 μl/min with aCSF beginning 30 min before asphyxia and continuing throughout the 2 h of reperfusion. The leukocyte adherence and vascular permeability data from these 2 asphyxia groups were statistically analyzed and graphed in comparison to their respective drug-treated groups, and the mean arterial blood pressure, blood glucose, arterial pH, arterial partial pressure of CO_2_, and arterial partial pressure of O_2_ data from these 2 asphyxia groups were also statistically analyzed in comparison to their respective drug-treated groups. In a similar fashion, the cranial windows of 2 separate non-asphyxial control groups were either superfused with aCSF or allowed to remain static once filled, and were imaged over the same time period. No difference in the magnitude of leukocyte adherence or vascular permeability to fluorescein was noted between these 2 control groups, so the data from the 2 non-asphyxial control groups were merged into a single control group.

### Drug Administration

Oxypurinol (5 mg/kg or 50 mg/kg) was dispensed as an intravenous bolus, 30 min before asphyxia. SOD (1,000 U/ml; Oxis International, Portland, OR) or C_3_ [pH 7.0, 300 μM; synthesized as described previously (27, 29, 35)] were dissolved in aCSF and administered by cortical superfusion (50 μl/min) through the cranial window starting 30 min before asphyxia and continuing throughout the 2 h of post-asphyxial reperfusion.

### Quantification of Intravascular Leukocyte Dynamics

Leukocytes adhering to the endothelium of pial venules were quantified during off-line videotape playback on a high resolution monitor by an observer blind to experimental condition using OPTIMAS image analysis software (BioScan, Inc., Edmonds, WA). Initially, the two-dimensional surface areas of 2 separate contiguous groups of 2 or more small (20–80 μm) post-capillary venules and their primary branches were determined. Then the three-dimensional area of the venous network was calculated by multiplying the determined area by π. Leukocytes were manually counted and designated as adherent if they remained stationary within the venule for longer than 10 s. The number of leukocytes per millimeter vessel surface was calculated by dividing the raw leukocyte number by the 3-dimensional area of the venular network.

### BBB Permeability

Intravenous sodium fluorescein (MW = 376) was used to assess the effects of different drug treatments on the increase in BBB permeability induced by asphyxia, as described in detail in earlier publications (32, 34). Briefly, after 2 h of reperfusion, 1 ml/kg of a 0.04% solution of sodium fluorescein was administered intravenously over 1 min.

Sodium fluorescein's extravascular presence was quantified using the OPTIMAS image analysis software with a linear intensity (0–255) scale. Video recordings were obtained just prior to the fluorescein injection and during the initial minute after its administration to obtain background and maximum/peak optical density (OD) values, respectively, and then again 20 minutes thereafter. The OD was measured offline in an experimental group-blinded manner at 3 separate extravascular locations along a large portion of the length of 2 different venules chosen from the video field vascular network (total of 6 perivenular locations). Changes in OD were normalized along a 0–100% scale based on the difference between the background OD and the maximum OD in each animal. The six perivascular OD values were typically similar, and were averaged to obtain a representative value for fluorescein leakage in the pial venular network for each animal.

### Statistical Analysis

Sample sizes were determined based on data obtained in a previous publication (32), using a power of 0.80 and an alpha value of 0.05. Sample sizes were determined to be *n* = 5 per group. Since a limited number of animals could be studied in 1 day and control and asphyxia animals were included with every drug-treated group, control and asphyxia groups have larger sample sizes than drug-treated groups.

**Figure 1:**
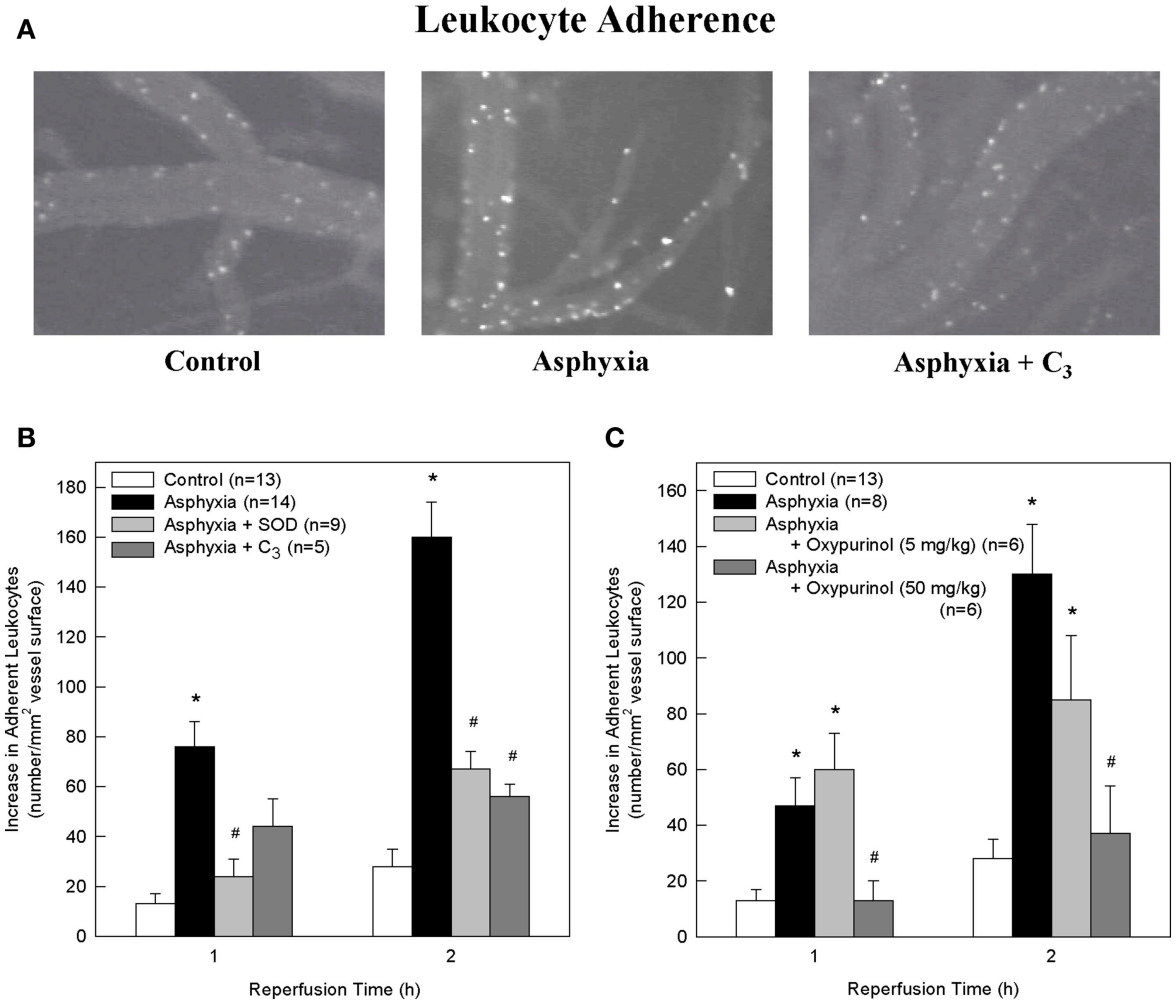
SOD, the SOD mimetic C_3_, and oxypurinol block the asphyxia-mediated increase in leukocyte adherence. **(A)** Representative images of rhodamine 6G-labeled adherent leukocytes in control animals, asphyxial animals, and asphyxial animals administered C_3_, at 2 h of reperfusion. **(B)** Animals induced with asphyxia and concomitant window superfusion with aCSF (*n* = 14) exhibited a statistically significant increase in leukocyte adherence relative to control animals (*n* = 13) at both 1 and 2 h of reperfusion. Animals continuously administered 1,000 U/ml SOD at a rate of 50 μl/min by window superfusion beginning 30 min prior to asphyxia (*n* = 9) exhibited significantly decreased leukocyte adherence relative to asphyxial animals at both 1 and 2 h of reperfusion. Animals continuously administered 300 μM C_3_ at a rate of 50 μl/min by window superfusion beginning 30 min prior to asphyxia (*n* = 5) exhibited significantly decreased leukocyte adherence relative to asphyxial animals at 2 h of reperfusion. **(C)** Animals induced with asphyxia without aCSF superfusion across the window (*n* = 8) had a statistically significant increase in leukocyte adherence relative to control animals (*n* = 13) at both 1 and 2 h of reperfusion. Animals administered 50 mg/kg oxypurinol 30 min prior to undergoing the asphyxia protocol (*n* = 6) exhibited significantly decreased leukocyte adherence relative to asphyxial animals at both 1 and 2 h of reperfusion. Animals administered 5 mg/kg oxypurinol 30 min prior to undergoing the asphyxia protocol (*n* = 6) exhibited no change in leukocyte adherence relative to asphyxial animals at either time point. Values shown (mean ± S.E.M.) represent increases above baseline in the number of leukocytes adherent to cerebral venules after 1 and 2 h of post-asphyxial reperfusion. *p < 0.05 vs. control; #*p* < 0.05 vs. asphyxia.

**Figure 2:**
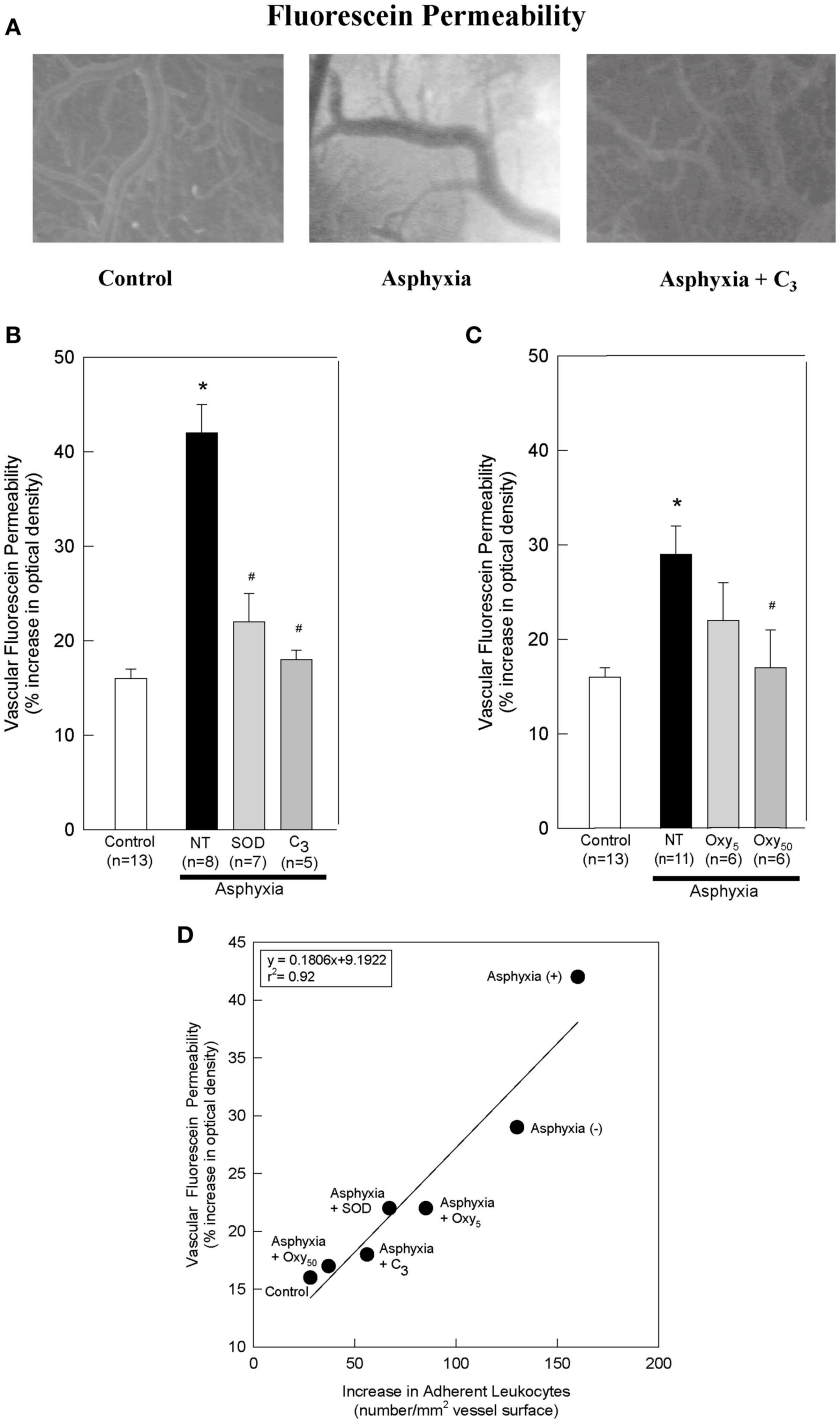
SOD, C_3_, and oxypurinol block the asphyxia-mediated increases in vascular fluorescein permeability, and vascular fluorescein permeability directly correlates with leukocyte adherence. **(A)** Representative images of sodium fluorescein permeability in control animals, asphyxial animals, and asphyxial animals administered C_3_, at 2 h of post-asphyxial reperfusion and 20 min after fluorescein administration. Raw images were used for all data analyses, but for publication clarity, images had brightness and contrast enhanced (all images modified equally). **(B)** Animals induced with asphyxia and concomitant cranial window superfusion with aCSF (NT; *n* = 8) exhibited a statistically significant increase in vascular fluorescein permeability relative to control animals (*n* = 13). Animals continuously administered 1,000 U/ml SOD at a rate of 50 μl/min by window superfusion (*n* = 7) exhibited significantly decreased vascular fluorescein permeability relative to asphyxial animals. Similarly, animals continuously administered 300 μM C_3_ at a rate of 50 μl/min by window superfusion (*n* = 5) exhibited significantly decreased vascular fluorescein permeability relative to asphyxial animals. **(C)** Animals induced with asphyxia without aCSF superfusion across the cranial window (NT; *n* = 11) exhibited a statistically significant increase in vascular fluorescein permeability relative to control animals (*n* = 13). Animals administered 50 mg/kg oxypurinol (*n* = 6) exhibited significantly decreased vascular fluorescein permeability relative to asphyxial animals. Animals administered 5 mg/kg oxypurinol (*n* = 6) exhibited no change in vascular fluorescein permeability relative to asphyxial animals. **(D)** A linear regression was generated by matching leukocyte adherence to the corresponding vascular fluorescein permeability under the different experimental conditions. Values shown (mean ± S.E.M.) represent the percent increase in optical density relative to that measured before fluorescein administration. NT, not treated; (+), with aCSF superfusion; (–), without aCSF superfusion. *p < 0.05 vs. control; #*p* < 0.05 vs. asphyxia.

All statistics were performed using SigmaStat 4.0. One-way analysis of variance (ANOVA) and Tukey tests were performed for data shown in Table 1 and for **Figures 2B,C**. Two-way repeated measures ANOVA and Holm-Sidak tests were performed for Figures 1B,C. A Shapiro-Wilk test was performed and a Spearman's rank correlation coefficient was determined for Figure 2D. Significant differences were noted if *p* < 0.05.

**Table 1:** Values are means ± S.E.M. Baseline values reported are averaged from two measurements recorded 30 min apart.

**Time point**	**Control**	**Asphyxia (+)**	**Asphyxia (−)**	**Asphyxia + SOD**	**Asphyxia + C_**3**_**	**Asphyxia + Oxypurinol (5 mg/kg)**	**Asphyxia + Oxypurinol (50 mg/kg)**
**MEAN ARTERIAL BLOOD PRESSURE (mm Hg)**							
Baseline	56 ± 1	57 ± 2	59 ± 1	55 ± 1	54 ± 1	55 ± 2	56 ± 1
1 h	55 ± 1	54 ± 2	58 ± 1	53 ± 1	51 ± 1	54 ± 2	55 ± 1
2 h	56 ± 1	52 ± 2	55 ± 1**^#^**	53 ± 0	52 ± 2	51 ± 1	54 ± 1
**BLOOD GLUCOSE (mg/dL)**							
Baseline	88 ± 7	104 ± 6	103 ± 5	95 ± 5	126 ± 3^*^	111 ± 22	91 ± 4
1 h	92 ± 7	120 ± 5^*^	117 ± 10	106 ± 8	135 ± 8^*^	112 ± 12	109 ± 10
2 h	93 ± 7	122 ± 6	107 ± 10	93 ± 10	124 ± 15	88 ± 10	89 ± 8
**ARTERIAL pH**							
Baseline	7.38 ± 0.01	7.36 ± 0.01	7.38 ± 0.01	7.34 ± 0.02	7.34 ± 0.02	7.41 ± 0.02	7.37 ± 0.02
1 h	7.36 ± 0.01	7.31 ± 0.02	7.32 ± 0.02	7.29 ± 0.02	7.29 ± 0.01	7.35 ± 0.02	7.33 ± 0.02
2 h	7.35 ± 0.01	7.35 ± 0.02	7.34 ± 0.02	7.33 ± 0.02	7.34 ± 0.02	7.40 ± 0.01	7.36 ± 0.01
**ARTERIAL PARTIAL PRESSURE OF CO**_**2**_ **(mm Hg)**							
Baseline	40 ± 1	38 ± 1	38 ± 1	38 ± 1	43 ± 1	37 ± 1	37 ± 2
1 h	41 ± 1	38 ± 2	40 ± 1	39 ± 1	41 ± 3	37 ± 2	40 ± 2
2 h	40 ± 1	35 ± 1	39 ± 1	39 ± 2	41 ± 2	36 ± 2	38 ± 3
**ARTERIAL PARTIAL PRESSURE OF O**_**2**_ **(mm Hg)**							
Baseline	94 ± 3	98 ± 3	95 ± 2	94 ± 3	88 ± 2	96 ± 3	91 ± 2
1 h	96 ± 2	101 ± 3	105 ± 4	103 ± 4	98 ± 4	93 ± 3	102 ± 4
2 h	92 ± 2	105 ± 4	100 ± 2	94 ± 2	112 ± 11^*^	94 ± 3	99 ± 4

*(+), with aCSF superfusion across the cranial window; (−), without aCSF superfusion across the cranial window*.

* p < 0.05 vs. control at the same time point;

# *p < 0.05 vs. baseline mean arterial blood pressure value for asphyxia without aCSF superfusion*.

## Results

Mean arterial blood pressure, blood glucose, arterial pH, arterial partial pressure of CO_2_, and arterial partial pressure of O_2_ at baseline and at 1 and 2 h of reperfusion are displayed in Table 1. Significant differences within and between groups are displayed in Table 1. While measures of blood glucose at 2 h, arterial pH at 1 h, and arterial partial pressure of CO_2_ at baseline and at 2 h demonstrated significant differences between the control, asphyxia with aCSF superfusion, asphyxia with SOD, and asphyxia with C_3_ groups overall (*p* < 0.05), *post-hoc* analyses revealed no individual between-group differences that were significant. No significant differences existed between the asphyxia groups and any of the corresponding treatment groups for any of the parameters at any time point.

Relative to non-asphyxial control animals, asphyxia resulted in progressive increases in the number of leukocytes adherent to cerebral venules during the initial 2 h of reperfusion (Figures 1A–C), consistent with our earlier studies (32, 34, 36). Adherence was 62% greater at 1 h and 23% greater at 2 h in asphyxial animals in which the window was superfused with aCSF (Figure 1B) relative to asphyxial animals in which the aCSF remained static during the experiment (Figure 1C).

After 2 h of post-asphyxial reperfusion, significant increases in sodium fluorescein permeability were also observed relative to non-asphyxial control animals (Figures 2A–C), indicative of a compromised BBB (32, 34, 36). In particular, in asphyxial animals with aCSF window superfusion, permeability was increased 42 ± 3% (Figure 2B), whereas in asphyxial animals with static aCSF under the window, permeability increased less (29 ± 3%; Figure 2C).

To determine if ${\text{O}}_{2}^{\underline{\cdot }}$ participated in promoting the elevated leukocyte adherence and/or the loss of BBB integrity that characterized the acute inflammatory response to asphyxia, we assessed the effects of removing ${\text{O}}_{2}^{\underline{\cdot }}$ by the extracellularly-confined enzyme SOD or by C_3_ (30). Topical superfusion of SOD significantly reduced leukocyte adherence at 1 and 2 h of reperfusion in asphyxial animals (Figure 1B). SOD also reduced asphyxia-induced elevations in BBB permeability to fluorescein (Figure 2B). Topical superfusion of C_3_ also reduced the post-asphyxial inflammatory response. As shown in Figures 1A,B and 2A,B, respectively, asphyxia-induced increases in both leukocyte adherence and BBB permeability were significantly attenuated by this ${\text{O}}_{2}^{\underline{\cdot }}$ scavenger.

We tested the hypothesis that xanthine oxidase is one source of ${\text{O}}_{2}^{\underline{\cdot }}$ following asphyxia by assessing the effects of oxypurinol on asphyxia-induced increases in leukocyte adherence and BBB permeability. Inhibition of endothelial and circulating xanthine oxidase activity with intravenous administration of oxypurinol significantly and dose-dependently reduced asphyxia-induced leukocyte adherence to post-capillary venules to levels that, at the higher dose, were not different from non-asphyxial controls (Figure 1C). Similarly, reductions in asphyxia-induced BBB breakdown were evidenced after administration of the higher dose of oxypurinol (Figure 2C).

When all experimental conditions were examined collectively, changes in leukocyte-endothelial adherence were highly correlated to changes in fluorescein permeability (*r*^2^ = 0.92; Figure 2D), suggesting a causal relationship between leukocyte adherence and BBB integrity.

## Discussion

We demonstrated a marked, time-dependent increase in leukocyte adherence to cerebral venules and a loss in BBB integrity during the initial 2 h of reperfusion following asphyxia. Local superoxide scavenging with C_3_ or SOD, or systemic inhibition of xanthine oxidase activity with oxypurinol, significantly decreased these inflammatory responses. These results suggest that the formation of inflammation-promoting concentrations of ${\text{O}}_{2}^{\underline{\cdot }}$ results in part from the breakdown of high energy adenine nucleotides and the resulting elevation in purine catabolites, which in turn serve as substrates for endothelial and circulating xanthine oxidase.

Numerous studies in the brain have demonstrated that oxygen free radicals are produced upon reperfusion following ischemia, and that their generation through various biochemical pathways during reperfusion is associated with increases in BBB permeability (37). The significant reduction in leukocyte adherence observed when animals were administered C_3_ or SOD indicates that ${\text{O}}_{2}^{\underline{\cdot }}$, generated from circulating neutrophils (38), cerebrovascular endothelial and smooth muscle cells (39), and/or the parenchyma surrounding the vasculature (38, 40–42), contributes to this vascular inflammatory response to asphyxia. The increase in BBB permeability following asphyxia is reduced by C_3_ or SOD treatment, which parallels the reduction in leukocyte adherence in asphyxial animals treated with either of these drugs. This is consistent with the notion that leukocyte adherence is a cause of BBB breakdown, at least in the acute post-asphyxial setting (43). Moreover, consistent with clinical studies of allopurinol in neonatal asphyxia (44), our results suggest that xanthine oxidase is a major source of ${\text{O}}_{2}^{\underline{\cdot }}$ produced in response to asphyxia, given that oxypurinol also significantly reduced asphyxia-mediated increases in leukocyte adherence and BBB permeability. The extent to which the latter inflammatory events result from oxypurinol-induced reductions in superoxide production and/or oxypurinol-induced increases in adenosine cannot be ascertained from the results of our study.

While all experimental treatments in this study were effective in reducing leukocyte adherence and BBB permeability, neither oxypurinol nor SOD is an ideal therapeutic compound. Oxypurinol first needs to be converted to an active state, and its potency is weak. SOD may lack substantial brain uptake due to its relatively large size. On the other hand, C_3_ has several features which may prove attractive clinically. It acts catalytically (27), and therefore retains activity for as long as it is present, has a plasma half-life of 8 h, and demonstrates significant brain uptake in both rodents and non-human primates (29, 45). C_3_ is also highly water soluble and has oral bioavailability (45). While C_3_ is roughly 100-fold less active than SOD itself (27), its ability to access mitochondria as well as other intracellular compartments may enhance its overall biological efficacy, especially as it appears to have a good safety profile with long-term (2 years, mice; 2 months, primates) chronic administration (19, 45). Newborn infants could theoretically be treated with C_3_ via oral fluids such as formula, or intravenously. Future investigation could help define the time window during which compounds such as C_3_ are effective in reducing the deleterious effects of neonatal asphyxia.

In this proof-of-concept study, we administered the superoxide scavenging agents C_3_ and SOD locally via cranial window superfusion, and the xanthine oxidase inhibitor oxypurinol systemically, 30 min prior to asphyxia, with the pharmacokinetics-based intention of providing some time for these agents to reach the intracellular and intercellular locations where superoxide might be generated rapidly during or shortly after asphyxia; whether their efficacy would have been as robust when administered after asphyxia, to more closely mimic the clinical situation, is unclear. We also did not explore the effects of oral or systemic C_3_ on post-asphyxial inflammation, as this would have involved dose-finding experiments requiring more C_3_ than was available. In addition, we focused on the acute response to asphyxia and did not attempt to quantify the extent of leukocyte diapedesis into brain parenchyma, nor measure endpoints, such as CA1 pyramidal neuron death, that are more indicative of asphyxial brain injury and the potential long-term neuroprotective effects of our interventions. Finally, while we found strong correlations between the extent of post-asphyxial leukocyte adherence to the venular endothelium and BBB breakdown under control conditions and in response to our interventions, we cannot conclude with certainty that the former caused 100% of the latter. It is possible that ${\text{O}}_{2}^{\underline{\cdot }}$ contributed to some portion of the observed BBB breakdown, not only independent of the extent of leukocyte adherence to the venular endothelium, but to BBB breakdown in other parts of the microvasculature as well.

In summary, we found that ${\text{O}}_{2}^{\underline{\cdot }}$ is produced in response to asphyxia, that xanthine oxidase is an important enzymatic source of this radical, and that scavenging of ${\text{O}}_{2}^{\underline{\cdot }}$ by administering C_3_ or SOD, or preventing its formation, can ameliorate this acute inflammatory response and lessen its potential to damage the neonatal cerebral microcirculation.

## Ethics Statement

This study was carried out in accordance with the recommendations of Public Health Service guidelines. The protocol was approved by the Washington University Institutional Animal Care and Use Committee.

## Author Contributions

JR wrote and revised substantial portions of the article. KQ prepared and tested C_3_ and wrote sections of the manuscript. EG and AS carried out the asphyxia experiments, including the fluorescent leukocyte labeling and imaging. TP contributed to initial experimental plans. NK contributed to writing portions of the article. LD and JG conceptualized the project, guided the experimental design and data analysis and interpretation, and contributed to writing portions of the article.

### Conflict of Interest Statement

KQ is currently employed by company PerkinElmer, but he was not affiliated with a commercial company at the time of data collection. The remaining authors declare that the research was conducted in the absence of any commercial or financial relationships that could be construed as a potential conflict of interest.
